# Is There a Relationship Between Medical Student Mistreatment and Specialty Choice and Career Intentions? A Systematic Review

**DOI:** 10.1007/s40670-025-02340-9

**Published:** 2025-02-26

**Authors:** Gursimrat Kaur, Kevin Peng, Rachel Urwin, Johanna I. Westbrook, Ryan D. McMullan

**Affiliations:** 1 Faculty of Medicine, Health and Human Sciences, Macquarie University, Sydney, Australia; 2 Faculty of Medicine, Health and Human Sciences, Australian Institute of Health Innovation, Macquarie University, Sydney, Australia

**Keywords:** Medical students, Mistreatment, Discrimination, Specialty choice, Career intentions

## Abstract

**Background:**

Negative experiences such as mistreatment during clinical placement can have implications for medical student specialty preferences. The aim of this study was to synthesise research on the relationship between medical student mistreatment and their specialty and career choices.

**Methods:**

We systematically searched five electronic databases (MEDLINE, Scopus, CINAHL, Embase, and PsycINFO) to identify studies published from database inception to June 2024 investigating the relationship between the mistreatment of medical students and their specialty choice and career intentions. Two reviewers independently screened articles for inclusion, assessed study quality, and extracted data. We performed a narrative synthesis of the included studies.

**Results:**

We identified 11 studies. The majority used a cross-sectional study design (*n* = 10). Seven were conducted in the USA. Mistreatment in the form of verbal abuse, discrimination, harassment, and bullying was reported by medical students across the studies. Seven of the studies found an association between mistreatment and specialty or career choices. General surgery and surgical specialties were the most hostile environments for clinical placement and deterred students from pursuing these specialties. Mistreatment was frequently experienced by medical students who identified as female or as a sexual minority. In obstetrics and gynaecology, male students reported experiencing more mistreatment than females and were consequently more likely to change their career choices.

**Conclusions:**

Our findings suggest that students are less likely to pursue specialties if they experience mistreatment during clinical training. Medical students were especially deterred from general surgery and surgical specialties due to their experiences of discrimination.

**Supplementary Information:**

The online version contains supplementary material available at 10.1007/s40670-025-02340-9.

## Introduction

The career choices of medical students are influenced by multiple factors, including student attributes and values, career expectations, medical school characteristics, and perceptions about different specialties [1–4]. A major component of medical education is delivered through clinical placements and experiences during these placements play an important role in determining student specialty preferences [3, 5–8]. One study found 70% of students based their decision of what specialty to pursue on their interactions with doctors during their placements [3]. Positive experiences on surgical placement are associated with an increased interest in pursuing surgical specialities in the future [5]. Equally, negative experiences during clinical placement, including mistreatment, influence the career choices of medical students [9].

Mistreatment includes behaviours that are inappropriate in the clinical teaching environment and range from incivility and bullying to sexual harassment and physical assault. The prevalence of mistreatment in medical training is high with a meta-analysis of 59 studies reporting 59% of medical trainees experienced at least one form of harassment or discrimination [10]. Mistreatment is associated with a range of negative implications for medical students that go beyond impaired learning including depression, suicidality, burnout, distress, and fatigue [11–15].

In a study of 1314 medical students from 14 medical schools in the United States, students who experienced gender discrimination or sexual harassment during clinical specialty placement were less likely to apply for those specialties for residency selection [16]. Students also question their choice of medicine as a career due to their experience of mistreatment. A US study found that medical students who reported experiencing mistreatment and discrimination in the first 2 years of medical school were significantly more likely to leave medical school [9]. Medical schools have a responsibility to develop a diverse medical workforce that aligns with the health needs of the population it serves. Mistreatment of medical students may exacerbate shortages in some specialties such as general surgery and paediatrics [17–19]. Historically, women are less likely to seek careers in surgery, orthopaedics, and urology, while fewer men pursue paediatrics and psychiatry [1]. Reducing these shortages and gender imbalances across specialties is important in increasing equal opportunities across specialties and delivering a diverse medical workforce to meet population needs.

There is currently no cohesive synthesis of study findings on the nature of the relationship between medical student mistreatment and specialty and career choice. To address this gap, we conducted a systematic review to examine this relationship and identify the specialties where mistreatment discourages students from pursing those specialties and careers. The identification of factors that deter medical students from choosing specialties is essential for medical schools to develop curricula, support systems, and learning experiences that optimally foster the next generation of medical doctors.

## Methods

Our systematic review was conducted in accordance with the Preferred Reporting Items for Systematic Reviews and Meta-Analyses standard [20].

## Search Strategy

One author searched Medline, Embase, PsycINFO, Scopus, and CINAHL for articles published from database conception to June 2024. We developed our search strategy with the aid of a clinical research librarian (Appendix A). Synonyms were linked together using the Boolean operators “OR” and “AND”. The results of the search were exported to Endnote Version 20 [21]. Duplicates were removed. References were then exported to Rayyan which is a web-based application that manages references and enables collaboration among team members and accessibility on multiple devices [22]. Two authors (KP, GK) independently screened titles and abstracts. Any conflicts were reviewed by an independent third author (RDM) who then had the final decision. The same two authors then examined full texts to screen for eligibility against the inclusion criteria. Any conflicts were reviewed and resolved by an independent third author.

## Inclusion Criteria and Exclusion Criteria

We included articles based on the following criteria: (a) original studies that reported a relationship between medical student mistreatment and specialty choice or career intentions; (b) studies published in a peer-reviewed journal; (c) studies published in English. Articles were excluded if study participants were not medical students (e.g. residents, registrars, junior doctors, non-medical personnel). Conference abstracts, reports, grey literature, and theses were excluded.

## Data Extraction

Two authors (KP, GK) extracted data on: first author, year of publication, country, study design, sample size, definition of mistreatment, qualitative and quantitative measurements, and key findings. Key findings were associations between mistreatment and specialty choice or career intentions. Discrepancies were resolved by consensus. Extracted data were compiled into a table using Microsoft Excel.

## Quality Assessment

The quality of included studies was assessed by two independent authors (KP, GK) using the Appraisal tool for Cross-Sectional Studies (AXIS) [23]. The AXIS tool was used to assess the quality of five areas: introduction, method, results, discussion, funding sources/ethical approval. The tool comprised a total of 20 ‘yes’, ‘no’, and ‘don’t know’ responses. Studies received 1 point for every ‘yes’ and 0 points for every ‘no’ or ‘don’t know’. The scores for each item were then summed to give an overall rating of the study with scores between 0 and 7 indicating a low-quality study, 8 and 14 a medium quality study, and 15 and 20 a high-quality study.

## Data Synthesis

We completed a narrative synthesis of the studies. Due to the heterogeneity of study design and outcome measures, we were unable to perform a meta-analysis.

## Results

**Fig. 1:**
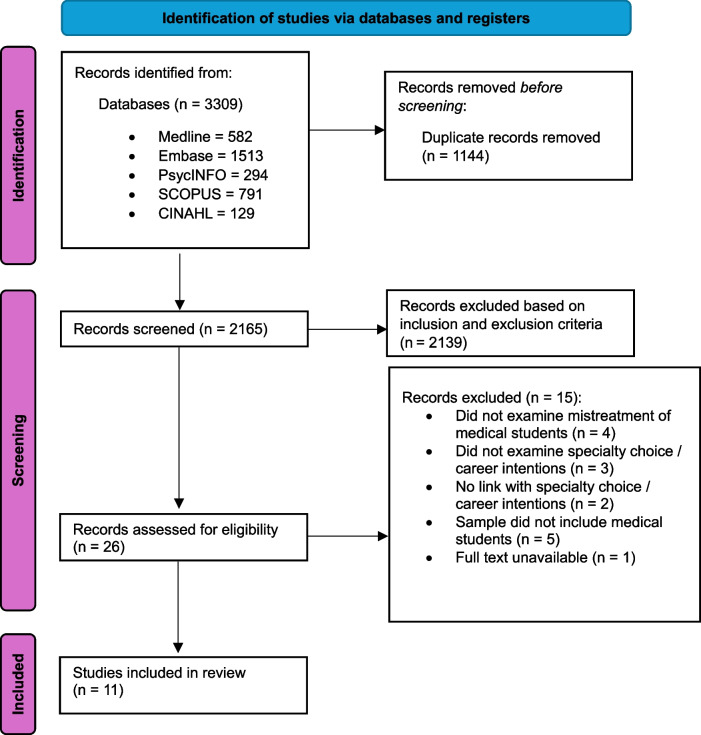
PRISMA flow diagram of the selection process

The literature search retrieved 3308 articles of which 1144 were duplicates. The title and abstracts of 2165 articles were screened. Of these, 26 articles were retained for full-text screening. In total, 11 articles met the inclusion criteria and were included in the review (Fig. 1). Seven studies were conducted in the United States (US) [16, 18, 24–28] and one each in Sweden [29], Canada [30], Poland [17], and Germany [31]. The year of publication ranged from 1990 to 2024. Ten studies were cross-sectional studies [16, 17, 24–31], and one was a longitudinal study conducted over an 8-year period [18]. All studies were of high quality: one study had an AXIS quality assessment score of 18 [28], seven studies had a score of 16 [16–18, 25–27, 31], and three studies had a score of 15 [24, 29, 30]. Study characteristics are outlined in Table 1.

**Table 1:** Study characteristics

First author, year, country	Study design	Number of participants	Definition of mistreatment	Measures	Key findings	Quality assessment
Haviland [28], 2011, USA	Cross-sectional	66,394 graduating medical students	Physical punishment, sexual harassment, psychological cruelty, and discrimination based on race, religion, ethnicity, sex, age, or sexual orientation	Association of American Medical Colleges’ 2000–2004 Medical School Graduation Questionnaire	At medical schools where higher percentages of senior graduating students were planning academic careers, students who reported experiencing mistreatment were less likely at graduation to be planning a career in academic medicine	18
Hunt [25], 1996, USA	Cross-sectional	1114 graduating students from nine medical schools	Badmouthing: Negative comments that students hear about their potential career choice. The comments are demeaning of the specialty	5-item questionnaire was used to examine the extent of negative comments	17% of respondents indicated they had changed their career choice due to badmouthing	16
Kristoffersson [29], 2018, Sweden	Cross-sectional	250 final year medical students from one medical school	No definition provided	A questionnaire comprising two open-ended questions asking respondents to describe events that made them interested or uninterested in specialities	Respondents indicated that excluding, hostile, and sexist workplace climates deterred them from specialities. This was the case more so for women (44%) compared to men (16%)	15
Madrigal [27], 2021, USA	Cross-sectional	383 enrolled medical students from all levels of undergraduate training at one university	Mistreatment examined was bullying and discrimination. No definitions provided	Questionnaire with four sections examining perceptions of pursuing various specialities and frequency of bullying and discrimination	Students who identified as a sexual or gender minority rated surgery and surgical subspecialties lowest on acceptance. They were less comfortable than other respondents when applying for residency in anaesthesiology and to emergency medicine	16
Nora [24], 1996, USA	Cross-sectional	169 senior medical students from two medical schools	Gender discrimination: those behaviours, policies, and other activities which adversely affect women because of disparate treatment, disparate impact, or the creation of a hostile environment. Sexual harassment: the use of authority to emphasise the sexuality or sexual identity of a person in a manner which prevents or impairs that person’s full enjoyment of educational benefits, climate, or opportunities	Questionnaire that examined student experience of gender discrimination and sexual harassment across specialities and whether their experiences influenced their specialty and residency programme choices	A majority of respondents indicated that their experiences of gender discrimination and sexual harassment did not influence their specialty and residency programme choices	15
Oser [18], 2014, USA	Longitudinal	801 third year medical students at one institution were surveyed over an 8-year period	Mistreatment: public humiliation, physical harm, verbal abuse, and various forms of coercion	A questionnaire examined the frequency and impact of mistreatment during clerkships and any mistreatment based on a student’s specialty choice	Approximately half of respondents believed they had to be less honest about their specialty choice to receive fair treatment. A majority of respondents did not change their interests in specialty from the beginning and end of the third year	16
Park [30], 2005, Canada	Cross-sectional	230 final year students at four medical schools	Discrimination. No definition provided	30-item questionnaire examined experiences in general surgery, planned residency specialty selection, and factors influencing career choice	Respondents who reported being deterred from general surgery were more likely to be women who experienced gender-based discrimination	15
Sheehan [26], 1990, USA	Cross-sectional	75 third year students at one medical school	Mistreatment, harassment, discrimination (racial and ethnic), and abuse (verbal, physical, and psychological)	A questionnaire examined the prevalence of mistreatment, harassment, discrimination, and abuse and the extent to which these behaviours influenced their attitudes towards medicine and their commitment to becoming a physician	37% of respondents indicated that they had considered dropping out of medical school due to mistreatment; 77% reported they were more cynical of the medical profession compared to when they started; and 24% indicated they would have chosen a different profession had they known about medical student mistreatment	16
Skorus [17], 2020, Poland	Cross-sectional	595 medical students from 11 medical schools	No definition provided	34-item questionnaire examined interest in pursuing a surgical career, lifestyle issues, surgical education, experience with the surgical environment, and the opinions of others	Negative comments regarding gender were not a risk factor for deciding against pursuing a surgical specialty	16
Stock [31], 2024, Germany	Cross-sectional	759 fifth and sixth year medical students from 31 medical faculties	Gender discrimination. No definition provided	34-item questionnaire examined the experience of gender discrimination and changes made to specialty choice	For 10.6% of women and 2.3% of men, gender discrimination was the reason why they changed specialty. Women more often reported gender discrimination as a reason for a change of a previously chosen specialty compared to men	16
Stratton [16], 2005, USA	Cross-sectional	1314 fourth year medical students at 14 public and private medical schools	Sexual harassment: the use of authority to emphasise the sexuality or sexual identity of a person in a manner that prevents or impairs that person’s full enjoyment of education benefits, climate, or opportunities. Gender discrimination: those behaviours, policies, and other activities, which adversely affect either women or men because of disparate treatment, disparate impact, or the creation of a hostile environment	A questionnaire was used to examine experiences of gender discrimination and sexual harassment during medical school and the extent to which their concerns about these behaviours influenced student choices of specialty and residency programme	Most students reported that considerations about gender discrimination and sexual harassment did not influence specialty choice or residency programme. However, a significantly greater proportion of women believed that gender discrimination and sexual harassment influenced their specialty choice and residency programme. Women were 2.2 times more likely than men to report that decisions about specialty choice were partly based on concerns about these behaviours; and 1.8 times more likely to report these behaviours influenced their residency programme rankings	16

## Verbal Abuse, Physical Abuse, Harassment, and Disrespectful Behaviours

Four studies investigated the effects of verbal abuse, physical abuse, harassment, or disrespectful behaviours on medical students [18, 26, 28, 29]. A US study of 75 third year medical students conducted in 1990 found that these behaviours impacted 37% of respondents who considered withdrawing from medical school [26]. Approximately 24% of respondents reported they would have considered an alternative profession had they known about the prevalence and severity of mistreatment in medical school. A second US study examined the relationship between student mistreatment and intentions to pursue academic medicine by analysing data from the Association of American Medical College’s Graduation Questionnaire comprising 66,394 responses [28]. Students who reported experiencing mistreatment at medical schools where academic medicine was the choice of a relatively high number of senior students were less likely to be planning careers in academic medicine at graduation.

A third study of 801 US medical students investigated the frequency and effect of mistreatment experienced by third year medical students over an 8-year period [18]. Although respondents to the questionnaire indicated that they experienced mistreatment during their clinical placements, a majority did not change their specialty choice at the end of their third year. Mistreatment was reported to occur most often during surgery, obstetrics-gynaecology, and internal medicine clerkships. A study of medical students (*n* = 250) in Sweden found 44% of females and 16% of males were deterred from specialties due to the workplace climate [29]. Workplace climate was the most common deterrent among women but was not ranked in the top three deterrents for men. Reasons for not choosing a specialty due to the workplace included experiences of hierarchy with senior physicians treating lower ranked colleagues poorly, male-dominated workplaces, and surgical wards where abuse of power, sexist jokes, and macho attitudes were common.

## Negative Comments About Career Choice

Two studies examined the association between negative comments and students’ specialty and career choices. The first study examined the prevalence and association between ‘badmouthing’ and career choices among 1114 US medical students across nine medical schools [25]. Findings indicated that 17% of respondents had changed their career choices after exposure to badmouthing. Badmouthing occurred most frequently for students selecting surgery and family medicine. In a study conducted across 11 Polish medical schools, negative comments related to gender and being discouraged from pursuing a surgical career by others were not associated with deciding against pursuing a surgical specialty [17].

## Gender Discrimination and Sexual Harassment

Five studies investigated the relationship between gender discrimination and sexual harassment and specialty choice [16, 24, 27, 30, 31]. In the first study, 21% of US medical students reported that gender discrimination and sexual harassment affected their specialty choice [24]. Eight per cent of male students who observed or heard about gender discrimination and sexual harassment reported that it had an impact on specialty and residency programme choice. Gender discrimination and sexual harassment were reported to frequently occur in general surgery and surgical specialties particularly for women. Men reported a higher prevalence of these behaviours in general paediatrics, paediatric subspecialties, and obstetrics-gynaecology. The second study conducted in the United States found 92% of women and 83% of men experienced, observed, or heard about at least one incident of gender discrimination and sexual harassment during medical school [16]. A majority of medical students (83% of men, 54% of women) reported that gender discrimination and sexual harassment did not influence their choice of specialty. However, a significantly greater proportion of women compared to men believed their specialty choice (${\chi }^{2}$ = 100.9, *df* = 1, *p* ≤ 0.0001) and residency programme rankings (${\chi }^{2}$ = 36.7, *df* = 1, *p* ≤ 0.0001) were influenced by gender discrimination and sexual harassment. Women were 2.2 times more likely than men to report that their specialty choices were in some part influenced by concerns about gender discrimination and sexual harassment. Women were also 1.8 times more likely than men to report that concerns about gender discrimination and sexual harassment impacted their residency programme rankings. Students specialising in obstetrics and gynaecology (43%), general surgery (21%), emergency medicine (20%), and paediatrics (19%) were most likely to report being exposed to gender discrimination and sexual harassment.

In a study conducted in Canada, 25% (*n* = 32) of final year female medical students who were deterred from general surgery experienced gender-based discrimination during their general surgery clerkships, compared to 3% of males [30]. The source of discrimination most frequently reported was surgical staff. A US study examined the impact of sexual and gender minority medical student experiences on career trajectory [27]. Respondents to the survey who identified as a sexual and gender minority (*n* = 103) were less comfortable compared to other respondents when applying for residency in anaesthesiology and emergency medicine. Attending physicians in general surgery and surgical specialties were perceived to be the least accepting of sexual and gender minority students. Two-thirds of the respondents had concerns that the disclosure of identity would affect their future career. The final study surveyed 759 medical students from 31 German universities [31]. Findings indicated gender discrimination was the reason why 10.6% of women and 2.3% of men changed specialty. The specialties with the most frequently reported gender discrimination included family medicine, surgery, internal medicine, orthopaedics, and gynaecology. The only specialty in which men experienced more discrimination than women was gynaecology.

## Discussion

The aim of this systematic review was to synthesise literature examining the relationship between medical student mistreatment and specialty and career choices. Across 11 studies, medical students reported experiencing various types of mistreatment during their clinical education. The behaviours experienced included verbal abuse (e.g. harassing, insulting, yelling at a student), physical abuse, negative comments (i.e. about specialties and career choices), discrimination, and sexual harassment. Seven of the 11 studies indicate a relationship between mistreatment and specialty choice and career intentions with students reporting they were less likely to pursue specialties if they had experienced mistreatment during clinical training. This was particularly common in specialties such as general surgery and other surgical specialties. Gender discrimination was frequently reported to influence specialty and career choices.

The types of behaviours reported as mistreatment in the studies ranged from negative comments and bullying to harassment and discrimination. Perceptions of what constitutes mistreatment can differ between students and staff [32]. The alignment among medical student, faculty educator, and hospital staff perceptions of what constitutes mistreatment is an essential step to ensure that inappropriate behaviours towards students are accurately identified and reported [33]. One way to do this is to use clinical vignettes and open discussion among participants to reach consensus on definitions of mistreatment [34, 35]. However, educating students and staff about mistreatment alone without significant investment from senior clinical staff to drive culture change will not be effective at reducing mistreatment.

Gender differences in workforces across specialties can be due to considerations related to work life balance, career goals, and salary renumeration [36, 37]. Factors related to fewer female medical students pursuing careers in trauma and orthopaedics include unsociable hours, technical aspects of surgery, and on-call commitments [38], while those that may deter students from neurosurgery similarly include stress, work-life balance, and inherent complexity of the specialty [39]. Our review demonstrates that mistreatment can also deter medical students from specialties such as general surgery, obstetrics and gynaecology, emergency medicine, and paediatrics. Women and sexual minority groups in particular are at higher risk of experiencing discrimination impacting their future in medicine.

Reported discrimination was commonly experienced in general surgery and surgical specialties. There is a declining interest in general surgery across countries with fewer students electing to complete surgical training [40, 41]. The mistreatment of students in surgical specialties could worsen this decline. These specialties must better support all students regardless of gender or other individual characteristics to allow students to pursue careers that interest them. Having a mentor is a significant contributor to medical students pursuing a surgical career [17, 42]. A more inclusive environment should be developed to nurture interest in surgical specialties and to provide students with support in mentor-trainee relationships and as part of formal mentoring programmes [43, 44].

Specialties including surgery were perceived by medical students to lack diversity and less accepting of gender and sexual minority students. Women remain underrepresented among surgical residents and practicing surgeons limiting the number of role-models for female students [45]. As of 2019, approximately 8% of orthopaedic surgeons in the United States were women [46]. Our review indicated that men were less likely to pursue obstetrics and gynaecology due to discrimination [16]. The mistreatment of male medical students may be another factor among several others including increased women entering medicine and patient gender bias that may account for the decline in the number of men entering obstetrics and gynaecology [47]. In the United States, 84% of residents in obstetrics and gynaecology in 2019 were female, representing a consistently increasing proportion in the previous decade [48]. Exposure to same-sex role models in specialties can influence decisions about specialty choice [49]. Female students choosing surgery are more likely to have attended medical schools that had same-sex role models [30, 50]. Addressing discrimination and promoting diversity with appropriate role-models in specialties such as surgery and obstetrics and gynaecology are required to ensure support for all medical students.

## Limitations

Findings from this review are limited by the quality of the included studies. Five of the studies were conducted over 20 years ago. A majority of the studies was conducted in the United States limiting the generalisability of the findings. Further studies are required to determine the extent to which mistreatment influences specialty choice in a similar way across countries. Ten of the 11 studies were cross-sectional surveys prone to responder bias as medical students who had experienced mistreatment may have been more likely to complete the surveys. However, the findings were consistent across studies and populations. Our review had several limitations. Our search was limited to English-language studies. Grey literature was excluded. It was not possible to perform a meta-analysis due to the heterogeneity of the studies.

## Conclusion

There is consistent evidence that medical student mistreatment is associated with specialty and career choice. The experience of discrimination deters medical students from pursuing specialties such as surgery, obstetrics, and gynaecology. Limitations of the literature suggest a need to conduct longitudinal studies and qualitative studies to gain greater insight into the relationship between mistreatment and specialty choice and the barriers that deter students from pursuing specialties. This research would better inform the development of targeted programmes to reduce the mistreatment of medical students and improve diversity across specialties.

## Supplementary Information

Below is the link to the electronic supplementary material.

Supplementary file1 (DOCX 15 KB)
