# Supplementary material for: Is There a Relationship Between Medical Student Mistreatment and Specialty Choice and Career Intentions? A Systematic Review
Source: Med Sci Educ. 2025 Feb 26;35(3):1777–86. doi: 10.1007/s40670-025-02340-9 (PMC12228901; doi:10.1007/s40670-025-02340-9)
Supplement: Supplementary file 1 — Supplementary file1 (DOCX 15 KB) [file 40670_2025_2340_MOESM1_ESM.docx]

Appendix A. Search Strategy for databases

Scopus

(“MD student*” or clerkship* or “MBBS student*” or “medical student*” or “medical training” or “clinical training” or “clinical placement*”)

AND

(mistreat* or abuse* or bully* or harass* or “negative treat*” or “negative comment*” or “negative* learn*” or aggress* or prejudice or sexism or embarrass* or discriminat* or discouragement)

AND

(((career* or specialty or speciality) W/2 (choice* or select* or aspir* or intent* or decision*)) or occupation* or speciali* or “medical profession” or vocation or “surgical training” or “surgical profession”)

Medline and PsycINFO

(MD student* or clerkship* or MBBS student* or medical student* or medical training or clinical training or clinical placement*).

AND

(mistreat* or abuse* or bully* or harass* or negative treat* or negative comment* or negative* learn* or aggress* or prejudice or sexism or racism or embarass* or discriminat* or discouragement).mp.

AND

(((career* or specialty or speciality) adj2 (choice* or select* or aspir* or intent* or decision*)) or occupation* or speciali* or medical profession or vocation or surgical training or surgical profession)

CINAHL

("Students, Medical") OR (MH "Education, Medical+") OR (MH "Education, Medical, Continuing") OR "(MD student* or clerkship* or MBBS student* or medical student* or medical training or clinical training or clinical placement*)

AND

("Verbal Abuse") OR (MH "Student Abuse") OR ("Bullying+") OR ("Sexism+") OR ("Gender Bias") OR ("Racism+") OR ("Embarrassment") OR ("Discrimination+") OR "(mistreat* or abuse* or bully* or harass* or negative treat* or negative comment* or negative* learn* or aggress* or prejudice or sexism or racism or embarrass* or discriminat* or discouragement).mp."

AND

("Student Selection") OR ("Career Planning and Development") OR ("Specialties, Surgical+") OR ("Specialties, Medical+") OR ("Specialization") OR ("Occupations and Professions+") OR "(((career* or specialty or speciality) adj2 (choice* or select* or aspir* or intent* or decision*)) or occupation* or speciali* or medical profession or vocation or surgical training or surgical profession)

EMBASE

(MD student* or medical clerkship* or MBBS student*or medical training* or clinical placement*)

AND

(mistreat* or abuse* or harass* or negative comment* or negative* learn* or aggress* or prejud* or sexism or embarrass* or discriminat* or discourage*)

AND

(career choice* or career select* or career aspir* or career intent* or career decision* or specialty decision* or occupation* or speciali* or medical profession or vocation or surgical training or surgical profession)
